# Invasive Spiders and Their Microbiomes: Patterns of Microbial Variation in Native and Invasive Species in Hawai'i

**DOI:** 10.1002/ece3.72175

**Published:** 2025-10-02

**Authors:** Madison J. Pfau, Sven Weber, Susan Kennedy, Henrik Krehenwinkel, George Roderick, Rosemary Gillespie

**Affiliations:** ^1^ Department of Ecology and Evolutionary Biology UC Santa Cruz Santa Cruz California USA; ^2^ Department of Environmental Science, Policy, and Management UC Berkeley Berkeley California USA; ^3^ Department of Biogeography Universität Trier Trier Germany

**Keywords:** biogeography, endosymbionts, gut microbiome, invasion biology, spiders

## Abstract

Invasive species can have detrimental impacts on the community structure and native species persistence, causing cascading impacts on ecosystem function. These effects are amplified in remote island ecosystems that are characterized by non‐representative and often diverse biota. The mechanisms behind successful invasions, particularly of arthropods, are varied, but growing evidence suggests that invasive species escape from their native predators and competitors. Recent research has suggested that gut microbiota can play an important role in arthropod fitness, with vertically transmitted endosymbionts and horizontally acquired microbes performing different functions. Here, we explored the extent to which the microbiome may facilitate the ability of spiders to exploit and ultimately adapt to novel environments. We examined co‐occurring pairs of native and invasive spiders across three locations in the Hawaiian Islands and compared them with mainland counterparts to test two core predictions: (1) gut microbiota would be shaped primarily by local environmental filters rather than invasion status, and (2) vertically transmitted endosymbionts would show stronger host‐specificity and reduced diversity in invasives. Using 16S rRNA amplicon sequencing, we found that the site explained 11.7% of gut‐microbial compositional variance compared to 6.5% for host species. These results suggest that each spider maintains a species‐specific level of α‐diversity but reassembles taxonomic composition according to local microbial pools, thus indicating high context dependence in environmental filtering. Invasive species were found to have a lower relative abundance of gut endosymbiont taxa, with one species, *Badumna longinqua* , showing little to no endosymbiont presence across sites, and the other, *Steatoda grossa* , exhibiting low but site‐specific abundance. We observed a strong localization effect, suggesting that these endosymbionts are also being acquired from local environments, not carried from ancestral ranges. These results suggest host–symbiont interactions have differential impacts on native and invasive species and that microbiota may facilitate the success of spiders in novel environments.

## Graphical abstract

We investigated how gut microbiota and endosymbionts influence the success of native and invasive spiders in Hawaiian and mainland environments. Gut microbial composition was shaped more by local environmental conditions than invasion status, whereas invasive species generally harbored fewer endosymbionts, with abundance varying by site. These findings suggest that environmental filtering and locally acquired symbionts may play a key role in invasive spiders' adaptation to new habitats.

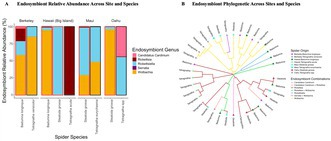

## 1 Introduction

Invasive species are one of the leading causes of biodiversity loss worldwide (Wilcove et al. 1998). Island ecosystems are particularly threatened by invasive species because of the high rates of endemism, trophic specialization, and low rates of predation for some species (Blumstein and Daniel 2005). Because of historical geographical isolation on islands, many species have developed highly specialized diets and microhabitats, which can be threatened by the introduction of exotic species (Pejchar and Mooney 2009; Novo et al. 2015). Although many factors contribute to the success of invasive species in novel habitats, a common feature is that they have escaped from population regulation by their native predators and competitors (Ni et al. 2021; Torchin et al. 2003). Recent work suggests the holobiont can facilitate invasion through its effects on digestion, pathogen resistance, and behavior (Abraham et al. 2017; Bredon et al. 2021), potentially influencing range expansion, diet generalization, reproductive success (Bonthond et al. 2021), and facilitating rapid adaptation (Łukasik and Kolasa 2024).

The properties conferred by microbial communities on their host differ depending on the nature of the association. Thus, gut microbiota, found in the gut lumen, tend to be dictated by the environment and diet and can affect digestion, nutritional state, and overall fitness (Turnbaugh et al. 2006; Shan et al. 2024; Pfau et al. 2023). In contrast, endosymbionts are intracellular (found in host cells or tissue) and often vertically transmitted, and hence may show phylogenetic patterns that mirror the host phylogeny (Perez‐Lamarque et al. 2022). Heritable endosymbionts can have large impacts on fitness, with some being shown to manipulate the outcome of host reproduction (Jiggins et al. 2002; Charlat et al. 2007; Hornett et al. 2008), impact host dispersal (in spiders: Goodacre et al. 2009), and change host immune function and development (in insects: Engel and Moran 2013; Lester et al. 2017). Although reproductive‐manipulation phenotypes have been noted in some spider–endosymbiont systems (Vanthournout et al. 2011; Gunnarsson et al. 2009), we still lack a comprehensive picture of how these intracellular associates shape spider ecology. Disentangling the contrasting roles of environmentally acquired gut communities versus vertically transmitted endosymbionts is therefore crucial for understanding the full spectrum of host–microbe interactions in spiders.

Gut microbes of arthropods—including spiders—are highly variable and generally horizontally acquired via diet or environmental exposure (Hu et al. 2019; Kennedy et al. 2020; Woodbury et al. 2013; Schmidt and Engel 2021). Debate remains over their functional impact: some argue high turnover precludes adaptive roles (Kennedy et al. 2020; Santos‐Garcia et al. 2020; Zhang et al. 2021), whereas others implicate the gut microbiome in metabolism (Ayayee et al. 2020), reproductive success (Shan et al. 2024), and toxin or pathogen resistance (Jaffar et al. 2022). Moreover, gut communities may facilitate invasion by helping hosts exploit novel diets and habitats (Lefort et al. 2023). As such, there is growing urgency to better understand how gut microbiome composition shifts across host species and ecological contexts.

Endosymbionts, however, exhibit more stable, host‐specific patterns. These symbionts are generally restricted to specific species and locations where they have been shown to manipulate reproduction, nutrition, and dispersion (Feldhaar 2011; Miller and Inouye 2012; Kobayashi and Hasegawa 2016). Endosymbionts may be facultative, meaning the host can survive without them, or obligate, meaning the host depends on them for survival (Weeks et al. 2007). However, spiders remain understudied in this context. Currently, some heritable symbiotic genera, including *Wolbachia*, *Rickettsia*, *Candidatus Cardinium*, *Serratia*, and *Rickettsiella*, have been documented in several spider families (Goodacre et al. 2009; Perez‐Lamarque et al. 2022; Mowery et al. 2024). However, some taxa appear to lack endosymbionts entirely (e.g., *Badumna longinqua* , Kennedy et al. 2020; *Latrodectus geometricus*, Mowery et al. 2024). Notably, both documented cases of endosymbiont absence involve invasive species, raising questions about how endosymbiont diversity and abundance vary with native versus invasive status. For instance, invasive brown widows ( *L. geometricus* ) have lost *Wolbachia* and *Cardinium* during range expansion (Mowery et al. 2024), and gray house spiders ( *B. longinqua* ) exhibit unusually low endosymbiont abundance (Kennedy et al. 2020). Thus, the ecological significance of endosymbiont absence, particularly in invasion success, remains understudied. To address this gap, we conducted comparative surveys of co‐occurring native and invasive spider species across multiple geographic regions to assess whether diversity patterns of gut microbes and endosymbionts are linked to invasion status and host species.

The current study aims to disentangle the relative roles of geography, host identity, and invasion status in structuring spider microbiomes, establishing a biogeographic baseline for how symbionts contribute to spider invasion dynamics. We examined three pairs of native and non‐native co‐occurring species on three different Hawaiian Islands: Oahu (native *Tetragnatha* sp. [Tetragnathidae; orb web]; non‐native *Steatoda grossa* [Theridiidae; tangle web]), Maui (native *Tetragnatha eurychasma* ; non‐native *S. grossa* ), and the Big Island (native *Tetragnatha acuta* ; non‐native *S. grossa* and *Badumna longinquia* [Desidae; cribellate sheet web]). All focal taxa are generalist predators but differ in web architecture, foraging mode, and microhabitat use. *Tetragnatha* species construct orb webs, typically in vegetation near freshwater or moist habitats, and capture primarily flying insects. *S. grossa* builds irregular cobwebs in sheltered crevices, preying on crawling arthropods and occasional vertebrates. *B. longinqua* produces sheet‐like funnel webs and often occupies human‐made structures and disturbed habitats. Although these structural and behavioral differences likely influence prey spectra and potential microbial exposure, detailed diet comparisons between native–invasive pairs remain lacking. To better assess commonalities among invasives from different regions, we added a fourth comparison between a native ( *T. versicolor* ) and non‐native ( *B. longinqua* ) species from California. Although several single‐species surveys have characterized spider microbiomes in some of these taxa (e.g., Kennedy et al. 2020 for *B. longinqua* ; Hu et al. 2019 for *S. grossa* ; Schmidt and Engel 2021 for *Tetragnatha*), no study to date has directly compared co‐occurring native–invasive host pairs in Hawai'i. Thus, we set out to extend on this previous work to assess commonalities among native and invasive species from different sites and regions.

For gut microbiota, we expected that composition would be dictated by a combination of the environment and prey diversity, with invasive species possibly harboring a higher diversity of microbes to enable a broader diet and improve environmental tolerance. We hypothesize that, for the gut microbiota, (1) environmental filtering will dominate such that native and non‐native species in the same area will show similar microbial abundance composition, and the same non‐native species from a different area will show a different microbial composition. We also expect that (2) overall gut microbial diversity (α‐diversity and β‐diversity) will not differ between native and invasive spider hosts sampled from the same site but will instead vary significantly among geographic locations and between host species. Specifically, we expect to observe higher diversity in native species in sites with distinct environmental microbial pools. For the endosymbionts, on the basis of previous findings in two different invasive species in different locations (Mowery et al. 2024; Kennedy et al. 2020), we predicted that (3) the endosymbiont diversity and abundance would be absent or at least lower in invasives compared to native species. Also, (4) endosymbionts will show similarities across populations of invasive species at different sites because of the recency of invasion; native spiders will exhibit phylogenetically coupled, multi‐lineage endosymbiont communities. We tested these hypotheses using DNA metabarcoding of the 16S ribosomal rRNA gene in five pairs of native and non‐native co‐occurring spider species at three different locations in the Hawaiian Islands and one in California.

## 2 Methods

### 2.1 Specimen Collection

We examined five pairs of native and non‐native co‐occurring species on three different Hawaiian Islands: Oahu (native *Tetragnatha* sp.; non‐native *S. grossa* ), Maui (native *Tetragnatha eurychasma* ; non‐native *S. grossa* ), and the Big Island (native *T. acuta* ; non‐native *S. grossa* and *B. longinqua* ). The native *Tetragnatha* species are part of a large adaptive radiation across the islands (Gillespie 2004). The non‐native *S. grossa* (native range: Palaeartic; Levi 1962) has been documented from the islands since 1900 (Simon 1900; Suman 1964), whereas *Badumna longinqua* (native range: Australia; Simó et al. 2011) is a more recent (1985) arrival (Roth and Nishida 1997). Ninety‐six spider specimens were collected from four locations (Table 1) over a 6‐month period, with Oahu specimens collected in January 2024, Maui specimens collected in December 2023, Big Island specimens collected in September 2023, and Berkeley specimens collected in March 2024. *Badumna longinqua* was collected from the Big Island and Berkeley. *Steatoda grossa* was collected from all sites except Berkeley, where *B. longinqua* was collected because of higher prevalence. Upon collection, specimens were taxonomically identified via morphology, sacrificed by freezing in a −800°C freezer, and preserved in 100% ethanol.

**TABLE 1:** Sample sizes of species collected at each site, including Oahu, Maui, and Big Island (Hawaii), and Berkeley (California).

Genus	Berkeley	Big Island	Maui	Oahu	Total
*Tetragnatha*	13	5	8	5	31
*Badumna*	10	18	0	0	28
*Steatoda*	0	7	14	7	28
*Scytodes*	0	0	0	5	5
*Thelacantha*	0	0	0	1	1
Total	23	30	22	18	93

### 2.2 DNA Extraction

To remove surface contamination, samples were soaked in a 1% bleach solution for 30 min, rinsed thoroughly in ethanol to remove bleach from the surface, and the opisthosoma (abdomen) was separated from the body with a sterile scalpel blade. We extracted DNA from the entire opisthosoma, which contains multiple organs but is dominated by the midgut. Recent studies using spider extractions have shown high microbial diversity in a spider's opisthosoma, with diversity concentrated in the gut lumen (Sheffer et al. 2019). Thus, we expect the entire microbiome to be well‐represented through the opisthosomal DNA extraction. The opisthosoma was transferred to a 1.5 mL screw cap tube and bead beaten at 2500 rpm for 1 min and 30 s. Upon successful homogenization of the material, samples were submerged in 300 μL of lysis buffer solution with 20 μL of Proteinase K and lysed at 55°C overnight.

DNA extraction was performed using the Puregene kit (Qiagen, Hilden, Germany), as described in De Kerdrel et al. (2020) and Krehenwinkel et al. (2022). Genomic DNA was precipitated using isopropanol and extracted using magnetic beads. Extracted DNA was eluted in 25 μL of water and quantified using a Qubit spectrophotometer (Fisher Scientific). DNA extracts were diluted to concentrations between 35 and 50 ng/μL for PCR amplification. Variable regions V1–V2 of the microbial 16S rRNA gene were amplified using the primer pair MS‐27F/MS‐338R (Donia et al. 2011) in 10 μL reactions and with 36 cycles using the Qiagen Multiplex PCR kit according to the manufacturer's instructions, with a 50°C annealing temperature. Upon confirmation of successful PCR via 1.5% gel imaging, a second round of dual‐indexing PCR (5 cycles) was performed to anneal 5′ tails consisting of an 8‐bp index and a P5 or P7 adapter, such that each final product had a unique combination of indexes (Lange et al. 2014) at a 56°C annealing temperature. Following amplification, indexed libraries were visualized again on a 2% agarose gel. Samples were then pooled into libraries on the basis of band intensity in equal amounts, cleaned using 0.8× AMPureBeads, and sequenced on an Illumina MiSeq using V3 chemistry. Negative controls for PCR and index PCR were sequenced in parallel to allow for subsequent removal of contamination.

### 2.3 Microbial Sequencing Analysis

The resulting sequence libraries were run through the QIIME2 (v. 2024.2) microbiome data science platform (Bolyen et al. 2019) for quality control, including removal of primer sequences using cutadapt (v5.0; Martin 2011), resulting in full sequences for each sample. Sequences were then filtered and denoised using Dada2 (Callahan et al. 2016). This resulted in a sample size of 105 total samples (including negative controls) post filtering, comprising a total of 755,737 16S rRNA reads. Amplicon Sequence Variants (ASVs) were assigned taxonomy using a naive Bayes GreenGenes taxonomy classifier trained on the SILVA database (Quast et al. 2013) with reference sequences clustering at 97% similarity. Assigned ASVs with fewer than five reads were removed from the dataset. All non‐bacterial sequences were removed, and classification, at least to the order level, was assigned to each ASV using a minimum similarity of 90%. Negative controls were included in feature table construction, and all probable contaminant sequences were removed from the tables before further processing by identifying all detected ASVs in the negative controls and removing all ASVs that had over 5% abundance in the negative controls relative to the other samples. The two most prevalent environmental contaminants were *Methylobacterium* and *Brachybacterium*. Following the removal of contaminant sequences, negative controls were pruned from the dataset for subsequent analysis. To equalize sampling depth, each sample was then rarefied to 1000 reads using the package “phyloseq” (McMurdie and Holmes 2013) to discard any that fell below this threshold in R version 3.5.1 (R Core Team 2014). This resulted in a final dataset of 93 specimens and 93,000 total reads, which was used for all downstream α‐ and β‐diversity analyses. ASVs were compiled into a table and analyzed using the package “phyloseq”. All future analyses were performed on relative abundance tables because amplicon sequencing depth varies and yields compositional count tables (i.e., the sum of counts per sample is fixed) (Gloor et al. 2017; McMurdie and Holmes 2013). Centered‐log ratio transformation was used to mitigate compositional constraints, but absolute abundance cannot be inferred.

### 2.4 Microbial Diversity Analysis

To test for separate effects on gut microbiota and endosymbionts, microbial data were subset for five known endosymbionts found previously in spider opisthosomas at the genus level: *Wolbachia*, *Rickettsia*, *Rickettsiella*, *Candidatus Cardinium*, and *Serratia*. We targeted these genera because two of these lineages (*Wolbachia* and *Rickettsiella*) have been shown to induce reproductive‐manipulation phenotypes in spiders (Goodacre et al. 2009; Rosenwald et al. 2020), and all five have been detected with variable prevalence in both our focal spider taxa and other spider species (Duron et al. 2008; Kennedy et al. 2020; Hu et al. 2019; Armstrong et al. 2021; Wang et al. 2024). Although selection of a limited number of endosymbionts may restrict our screening of all potential heritable partners, we believe the above‐chosen symbionts act as an effective representation of the endosymbiotic community. The remaining genera were considered gut microbiota. Microbial richness, as measured by Shannon's diversity index, was calculated for each spider specimen as a measure of α‐diversity. To evaluate the effects of geographical location, host identity, and specimen type on α‐diversity, a linear model was fitted with main effects of Island, Species Identification, and Specimen Type, plus the Island × Specimen Type interaction (the only interaction supported by our unbalanced sampling). Significance was assessed via Type III ANOVA (car v3.1‐3; Fox and Weisberg 2019) to properly handle unequal cell sizes.

Community β‐diversity was quantified as pairwise Bray–Curtis dissimilarities on relative‐abundance data (phyloseq). We ran PERMANOVA (adonis2, vegan) with main effects of Site, Species Identification, and Specimen Type, plus the Site × Species interaction (three‐way and other two‐way interactions were omitted because of unbalanced sampling). Post hoc pairwise contrasts (e.g., Hawaii vs. Berkeley; Steatoda vs. Tetragnatha [all species]; Tetragnatha [all species] vs. Badumna) were conducted via pairwiseAdonis with Benjamini–Hochberg adjustment and checked with ANOSIM (vegan, 999 permutations). Group‐level separation of microbial community composition among islands, host taxa, and specimen types was visualized using NMDS (metaMDS, vegan).

### 2.5 Relative Abundance of Microbial Taxa in Gut Microbiota and Endosymbionts

Relative abundances were calculated by applying a total‐sum scaling transformation: each ASV count within a sample was divided by the total number of reads for that sample. This transformation was applied to the cleaned dataset to generate a relative abundance phyloseq object. Across the two datasets, composition plots for each location and across each species were generated using the “microbiome” package in R (v. 1.26.0; Lahti et al. 2017).

#### 2.5.1 Gut Microbiota

To determine which microbial taxa were driving variation, the sites and species contributing to significant group differences were identified using pairwise PERMANOVA tests across site and species identity. These tests used distance matrix values from gut microbiota data as the response variable and were conducted using the R package *pairwiseAdonis* (v. 0.4.1; Martinez Arbizu 2020). Pairwise comparisons were conducted for: (1) species identity (*Steatoda* vs. *Tetragnatha*, *Tetragnatha* vs. *Badumna*), (2) native vs. non‐native classification, and (3) location (e.g., Hawaii vs. Berkeley, Maui vs. Berkeley, Oahu vs. Berkeley). Adjusted *p*‐values were calculated using the Benjamini–Hochberg correction to control for multiple testing. Finally, to determine which genera were driving differences across sites and species, SIMPER analysis was conducted using the R package *vegan* to identify which ASV relative abundances were associated with site and species identity, on the basis of the top 10 genera with the highest relative abundance (Oksanen et al. 2020). Given that our results indicated that a large portion of taxa were in low‐prevalence, we also included a top 15 genera figure to illustrate a larger range of taxa. Only contributions with a *p*‐value below 0.01 and a functional contribution greater than 4% were included. Stacked bar plots were used to visualize the composition of endosymbionts and gut microbiota (top 10 genera) at the genus level across locations and species using “ggplot2” (Wickham 2016). In parallel, we looked at the Indicator Species Analysis (ISA) to identify endosymbiotic microbial taxa significantly associated with specific species or locations. Significant indicator taxa were determined on the basis of IndVal scores using indicator species, with a significance threshold of *p* < 0.05. Hierarchical clustering of gut microbiota composition was performed using average linkage, visualized with *ggtree* (Yu et al. 2017), and statistically supported with *pvclust* (Novikov 2019). Differential abundance analysis was conducted using DESeq2 (Love et al. 2014), supplemented with SIMPER and ISA to highlight taxa most strongly associated with specific hosts or locations.

As a post hoc analysis to improve potential interpretations, we used *PiCRUSt2* (Douglas et al. 2020) to infer the potential functions of the top 10 most abundant gut microbes, which maps functional descriptions from 16S amplicon sequences to ASV and allows direct comparison to reference genomes. We used the PICRUSt2 v2.6.0 reference database (Douglas et al. 2020), which contains ~20,000 bacterial and archaeal genomes from the JGI IMG database. The full list of genomes and associated accession/citation metadata is available in the PICRUSt2 GitHub repository (https://github.com/picrust/picrust2) or upon request. Stratified *PiCRUSt*2 values were combined with aligned feature ID taxonomic assignments to identify microbe type to functional abundances and merged annotated descriptions of functional pathways using the MetaCyc pathway (EC accessions; Caspi et al. 2019). The top 10 most abundant taxa were identified using the relative abundance of the taxa to subset the functional descriptions for each microbe, and the top 10 most abundant functions per microbe were identified using the functional relative abundance scores.

#### 2.5.2 Endosymbionts

To determine the association between endosymbiont infection status and host or environmental factors, generalized linear mixed models were used with a binomial error distribution. For each endosymbiont genus (*Wolbachia*, *Rickettsia*, *Rickettsiella*, *Candidatus Cardinium*, and *Serratia*), binary infection variables (presence/absence per specimen) were created and modeled using the glmmTMB package (Brooks et al. 2017). Predictors included site, spider species identity, and native versus non‐native classification. Model fit was assessed using simulated residual diagnostics from the DHARMa package (Hartig 2024). Infection rates were also calculated as the proportion of individuals infected per species and island for each symbiont genus. We classified genera present in ≥ 30% of individuals as likely persistent, host‐associated symbionts and those below this threshold as potentially transient or prey‐derived—a cutoff that falls within the 22.8%–32.4% infection range reported by Duron et al. (2008) and aligns with common core–microbiome occupancy thresholds (Risely 2020). These analyses provided evidence for distinguishing potential persistent symbionts from transient microbial signals. To further support these interpretations of infection across sites and species, we examined species‐level consistency for each endosymbiont genus by identifying the number of unique ASVs per species‐site combination.

## 3 Results

After filtering for quality assurance, the final dataset yielded 1,045,238 reads across 96 samples, with a total of 6547 ASVs. Because of the largely unexplored microbiome of Hawaiian arthropods, most ASVs were assigned above the genus level. Host spider species' microbial assemblages were unique, with *B. longinqua* containing 21.7% unique ASVs, *S. grossa* with 29.1%, and *Tetragnatha* spp. with 37.2%, totaling to 88% of all ASVs not being shared across species (Figure S1A). Sites also experienced high specialization of ASV distinctiveness, with a total of 91.5% of ASVs coming from a single location, with Oahu containing 24.3% of unique ASVs, Maui with 24.5%, the Big Island with 23.2%, and Berkeley with 19.7% (Figure S1B).

### 3.1 Environmental Filtering Dominates Gut Microbiota

#### 3.1.1 Differences in Gut Microbiota Composition Reveal Environmental Filtering (H1)

**FIGURE 1:**
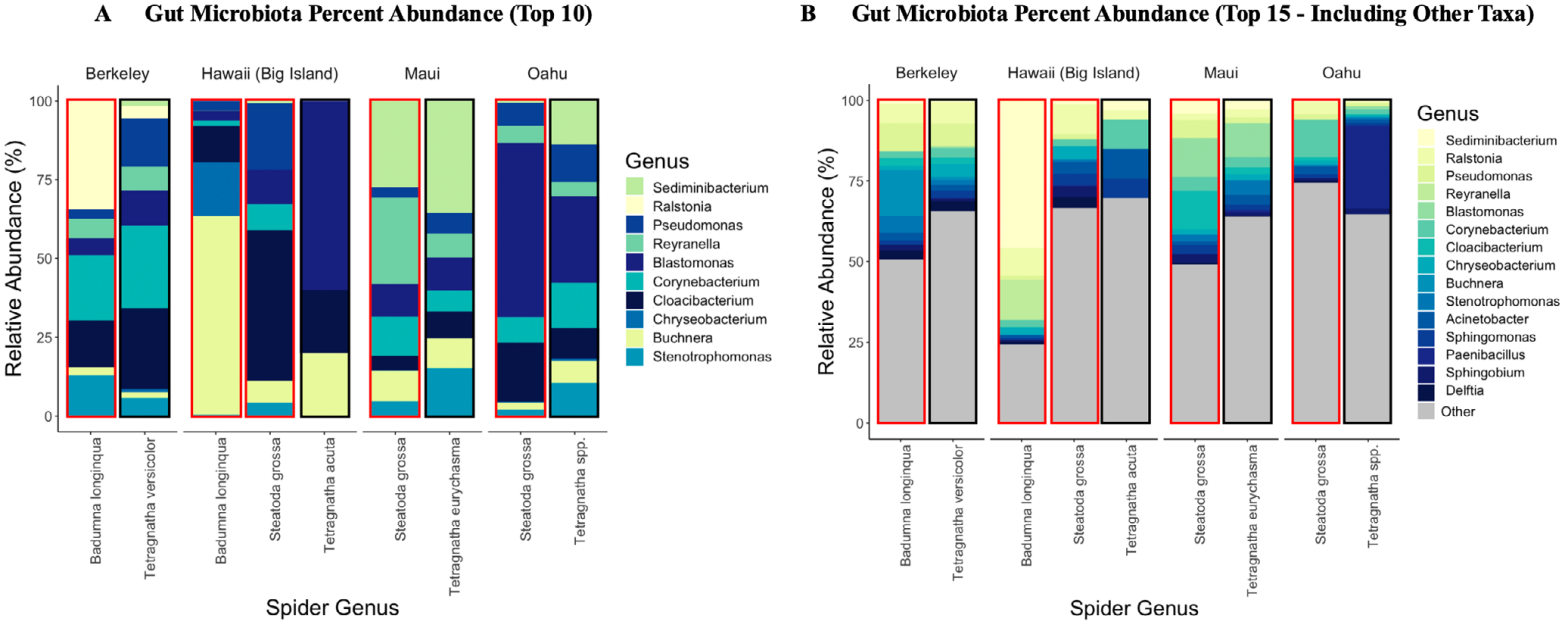
Variation in relative abundance across sites and species identifications, with microbial endosymbionts and gut microbiota separated. Black borders indicate species, whereas red borders indicate species. (A) Visualizes data using the percentage of abundance to 100%, excluding all non‐top 10 taxa. (B) Showcases the percentage of abundance including “other” categories, which account for all taxa not in the top 15 most abundant taxa, indicating that the top 10 highest abundant only account for a small portion of the total microbiome.

Across sites but within species, the top 10 gut microbiota were detected and categorized. *B. longinqua* exhibited a dominance of one particular genus, *Sediminibacterium*, on Big Island sites, whereas *S. grossa* and *Tetragnatha* spp. exhibited more equal distributions of microbiota (Figure 1). Because a substantial fraction of gut ASVs fell outside the top 10 most abundant taxa, Figure 1B includes five extra low‐frequency taxa, highlighting that the spider microbiome is dominated by many rarer ASVs.

### 3.2 Differences Between Native and Non‐Native Species in Gut Microbiomes

#### 3.2.1 Microbial Drivers of Compositional Differences (H1)

PERMANOVA tests were used to understand what sites contributed to dissimilarity across sites and species. Site explained 11.7% of the variance (*F* = 4.28, *p* < 0.001), species identity 6.5% (*F* = 3.60, *p* < 0.001), and specimen type (invasive vs. native) 0.9% (*F* = 0.95, *p* = 0.672), with 81% residual. ANOSIM supported that site and species were the highest predictors of group separation (Island *R* = 0.298; Species *R* = 0.468; Specimen *R* = 0.284; all *p* < 0.001; Table S3). Supplementary differential abundance analysis using DESeq2 supported this finding, identifying 19 significantly differentially abundant taxa between *Steatoda* and *Badumna*, and 18 between *Tetragnatha* and *Badumna*. Environmental comparisons also exhibited a strong influence on microbial composition with 17 significantly different ASVs in Hawaii vs. Berkeley, 13 in Maui vs. Berkeley, and 12 in Oahu vs. Berkeley. Together, these results indicate that biogeographic factors were the strongest structuring force in these gut communities, with host species identity also contributing meaningfully to microbial differentiation.

These results also prompted a post hoc SIMPER analysis to test which microbes were driving differences in correlations among sites and species, which demonstrated that the microbes contributing the most to differences were from *Sediminibacterium* spp. and *Propionibacterium* spp. overall, with the Big Island exhibiting the greatest variation from other sites and Berkeley, Oahu, and Maui all contributing to variation in *Propionibacterium* spp. abundance (Table 2). Species‐specific analysis indicated that the highest contribution to variance was from *Sediminibacterium* and concentrated for *B. longinqua* correlations, indicating that across species and sites, *Sediminibacterium* is important in driving differences in gut microbiota relative abundance. SIMPER analysis revealed that specific microbes have a disproportionate effect on site‐ and species‐specific changes in microbial relative abundance. In addition, ISA was used in combination with SIMPER and DESeq2 to identify taxa uniquely associated with specific species and locations, supporting our post hoc analysis. Notably, *Sediminibacterium* was significantly enriched in *B. longinqua* . Island‐level analyses revealed that Hawaii hosted a unique set of microbial taxa distinct from Berkeley. Among the most enriched taxa in species comparisons were those exhibiting log_2_ fold changes of 24.73, 22.94, and −24.71 in the *Steatoda* versus *Badumna* comparison, and −25.29 and −12.12 in the native versus non‐native comparison. In contrast, environmental comparisons, particularly Big Island versus Berkeley, showed even more pronounced enrichment patterns, with the top 10 most abundant taxa displaying log_2_ fold changes of 27.19, 18.69, and 15.74. These results support the conclusion that both host context dependence and environmental factors play a dominant role in shaping microbial composition in these spider species.

**TABLE 2:** SIMPER analysis results showing the average contribution of each taxon at the genus level to each tested correlation according to the pairwise Adonis test with significance codes (*** *p* < 0.001, ** *p* < 0.01, * *p* < 0.05).

Tested correlation	Average cont.	SD	AVA	AVB	C. Sum	*p*	Gut microbiota genus
Site							
Oahu × Maui	7.70%	0.01	0.00154	0.05094	0.2171	0.002**	*Blastomonas* spp.
Big Island × Maui	6.95%	0.099	0.00078	0.05094	0.2438	0.001***	*Blastomonas* spp.
Maui × Berkeley	8.06%	0.109	0.05094	0.00064	0.088	0.001***	*Blastomonas* spp.
Big Island × Oahu	5.74%	0.066	0.0465	0.00011	0.3218	0.012**	*Reyranella massiliensis*
Big Island × Berkeley	5.87%	0.067	0.0465	0.00037	0.2492	0.001***	*Reyranella massiliensis*
Big Island × Maui	5.29%	0.061	0.0466	0.0003	0.3005	0.002**	*Reyranella massiliensis*
Big Island × Oahu	16.90%	0.161	0.1338	0.00155	0.1784	0.004**	*Sediminibacterium* spp.
Big Island × Berkeley	17.10%	0.171	0.1338	0.0001	0.1856	0.001***	*Sediminibacterium* spp.
Big Island × Maui	15.70%	0.14	0.1338	0.0112	0.1688	< 0.001***	*Sediminibacterium* spp.
Species identification							
*B. longinqua* × *T*. spp.	15.30%	0.147	0.08	0.00119	0.1746	0.001***	*Sediminibacterium* spp.
*B. longinqua* × *S. grossa*	12.00%	0.113	0.08	0.0044	0.1419	0.001***	*Sediminibacterium* spp.
*B. longinqua* × *T*. spp.	4.72%	0.046	0.0255	0.00019	0.4249	0.001***	*Reyranella massiliensis*

*Note:* Average cont. is the average contribution of the taxon in percentage, SD is standard deviation, AVA is average contribution to dissimilarity, AVB is average dissimilarity between two groups, C. Sum is cumulative contribution of the taxon to all associations, *p*‐value is the significance of the contribution (only significant associations are shown here, and significant results are bolded for clarity).

Our *PICRUSt*2 analysis inferred potential functional characteristics in METACYC gene pathways of most of the abundant taxa (Figure S2). A majority of microbes were associated with enzymes that perform cellular maintenance functions, such as respiration and cell–cell signaling, but we also observed functions associated with metabolism, inflammation regulation, and more specific functions, such as biosynthesis of leucine and detoxification properties. Functional abundance differed across taxa, with *Sediminibacterium* spp. and *Blastonomas* spp. exhibiting the highest functional abundances, corresponding to the relatively high abundance of both taxa in the gut (Figure S2). However, because of the lack of strong functional inference, these functional assignments should be viewed as provisional hypotheses requiring metagenomic validation.

### 3.3 Diversity of Gut Microbiota

#### 3.3.1 Similarities in Diversity Despite Taxonomic Turnover Differences in α‐ and β‐Diversity (H2)

**FIGURE 2:**
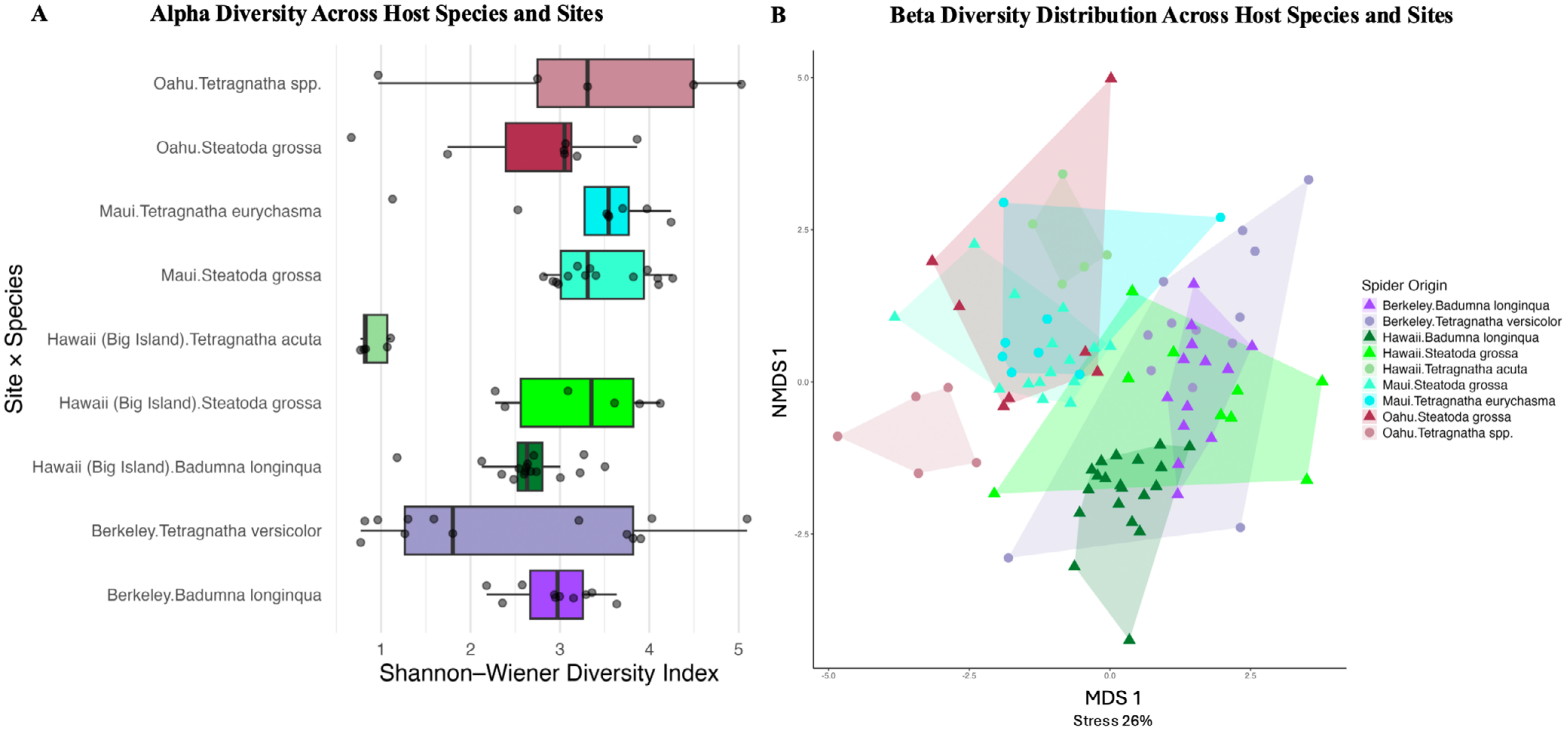
Visualizations of diversity analysis of gut microbiota. (A) α diversity does not differ across sites and species except for Big Island Tetragnatha. (B) β diversity NMDS plot visualizes dissimilarity across site × host genus, which indicates low clustering across sites and species. Triangles emphasize invasive species, and points depict native species for the respective locality.

When selecting only for gut microbiota and excluding endosymbionts, the microbial community α‐diversity (as measured by Shannon–Wiener Diversity Index) varied among species (Type III ANOVA: *F*_5,74_ = 3.42, *p* = 0.0078), whereas island (*F*_3,74_ = 1.21, *p* = 0.313) and native vs. invasive status (*F*_1,74_ = 0.36, *p* = 0.553) did not significantly influence α‐diversity. Because of unbalanced sampling design, we ran a secondary model to assess interactions among Island and native vs. invasive status, which revealed that island (*F*_3,76_ = 8.48, *p* < 0.001) and the interaction term (*F*_3,76_ = 4.93, *p* = 0.0035) became highly significant, indicating that the effect of invasive vs. native status on diversity depends on location; status alone remained non‐significant (*p* = 0.246). One‐way ANOVAs performed within each island showed that only the Big Island exhibited significant species‐level differences in Shannon diversity (*F*_2,24_ = 26.39, *p* = 8.7 × 10^−7^) (Figure 2A).

**FIGURE 3:**
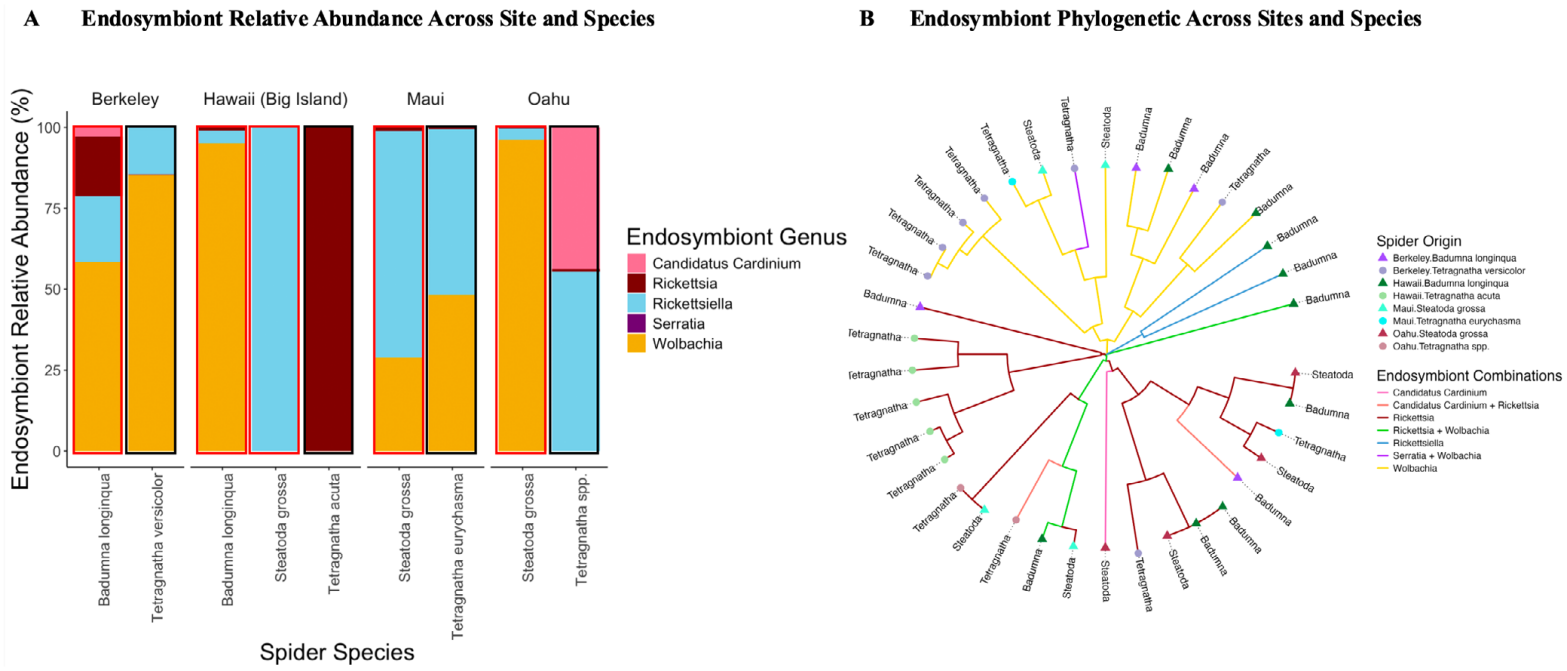
(A) Variation of non‐gut endosymbionts in relative abundance across sites and species identifications, with all specimens in the same assignments pooled together. Endosymbiont microbial genus assignments of relative abundance expressed as percentages for each host species within a particular site. Red borders indicate invasive species hosts, and black borders indicate native species hosts. (B) Endosymbiont phylogeny analysis on the basis of Bray–Curtis dissimilarity across sites and species, showing similarity among species at particular locations.

PERMANOVA was used to quantify the proportion of variance in gut microbial composition explained by collection site, species identity, and specimen classification. Island was the strongest predictor (*R* ^2^ = 0.117, *F* = 4.51, *p* = 0.001), followed by species identity (*R* ^2^ = 0.065, *F* = 3.78, *p* = 0.001). The Island × Species interaction was also significant (*R* ^2^ = 0.066, *F* = 2.54, *p* = 0.001), indicating that species‐specific differences in microbial composition vary by location. In contrast, specimen classification (native vs. non‐native) was not significant (*R* ^2^ = 0.009, *F* = 0.996, *p* = 0.545). These results support the hypothesis that environmental context plays a stronger role than host identity or native status in shaping gut microbial communities, and further suggest location‐specific filtering of microbial taxa within species (Figure 3B). Pairwise PERMANOVA comparisons supported this pattern: differences between *Badumna* and *Steatoda* (*R* ^2^ = 0.097, *F* = 6.64, *p* = 0.001), and *Badumna* and *Tetragnatha* (*R* ^2^ = 0.094, *F* = 6.51, *p* = 0.001) were stronger than those between *Steatoda* and *Tetragnatha* (*R* ^2^ = 0.027, *F* = 1.69, *p* = 0.001), suggesting more pronounced divergence between invasive and native species than among native species alone.

### 3.4 Reduced Endosymbiont Diversity and All‐or‐Nothing Dominance

#### 3.4.1 Differences in Endosymbiont Relative Abundance Across Sites (H3 and H4)

When only selecting for endosymbionts within the dataset, the community indicated significant variation among sites, with less consistent differences among species or between native vs. invasive status (Figure 3A). Though not all *Tetragnatha* species carried the same symbionts, members of this genus collectively hosted four of the endosymbiont genera detected in our dataset (*Wolbachia*, *Cardinium*, *Rickettsia*, and *Rickettsiella*), suggesting broader symbiont prevalence in native spiders. In contrast, invasive species ( *B. longinqua* and *S. grossa* ) exhibited low abundances of endosymbionts overall. *B. longinqua* carried only two genera, *Wolbachia* and *Rickettsiella*, both at very low relative abundance and with low consistency across individuals. Because the spiders were wild caught and fed on a wide range of taxa, it is possible that these few cases may reflect endosymbionts in the prey rather than the spider. Our binomial GLMMs (Table 3) show significantly lower Wolbachia prevalence on the Big Island, elevated *Rickettsiella* prevalence on Maui and in Steatoda, and higher *Rickettsiella* odds in Hawaiian Tetragnatha versus Badumna; Cardinium was uniformly rare with only *Tetragnatha* spp. (found on Oahu) predicting abundance. Applying our 30% prevalence cutoff, *Wolbachia* and *Rickettsiella* qualified as core symbionts in most host–site combinations (e.g., *Wolbachia* ≥ 50% in several populations; *Rickettsiella* = 100% on Maui and Oʻahu), whereas *Cardinium* and *Rickettsia* never exceeded 29%, supporting that they may be transient or prey‐derived (Table S1). ASV richness per genus remained low and dominated by a single ASV, though Big Island Tetragnatha occasionally harbored additional ASV diversity (Table S2).

**TABLE 3:** Significant predictors of endosymbiont presence across islands and species from binomial GLMMs.

Model	Predictor	Estimate	Std. error	*z*	*p*
Island‐based models					
*Wolbachia* presence	Island (Big Island)	−1.8233	0.7186	−2.537	**0.0112**
*Rickettsia* presence	Island (Big Island)	1.6337	0.8441	1.935	0.0529
*Rickettsia* presence	Specimen type (Non‐native)	−1.3029	0.6488	−2.008	**0.0446**
*Rickettsiella* presence	Island (Maui)	4.644	1.06	4.382	**0.001**
Species‐specific models					
*Cardinium* presence	Tetragnatha spp.	2.813	1.369	2.055	**0.0398**
*Rickettsiella* presence	*Steatoda grossa*	3.2896	0.7688	4.279	**0.001**
*Rickettsiella* presence	*Tetragnatha eurychasma*	3.9828	1.2327	3.231	**0.0012**

*Note:* Species‐specific models use *Badumna longinqua* as the reference point of comparison, and island‐based models use Berkeley as the reference point of comparison. *B*‐values represent log‐odds (logit scale), and SE is the standard error. Significant results are bolded.

#### 3.4.2 Low Similarity Across Invasive and Native Species (H4)

Phylogenetic analysis of endosymbiont ASVs revealed broadly intermingled lineages across both islands and host taxa, with a potential tendency for Big Island *T. acuta* ASVs to cluster more tightly and for Berkeley *Tetragnatha* ASVs to harbor multiple, phylogenetically distinct ASV clades (albeit with more internal variation). This pattern suggests that, whereas endosymbiont communities are largely shared across hosts and locations, there may be weak, host‐ or site‐associated grouping within the *Tetragnatha* genus. Moreover, invasive spider microbiomes failed to form distinct clusters across sites, likely reflecting an absence of phylosymbiosis. (Figure 3B).

## 4 Discussion

Understanding how microbial communities differ between native and invasive species provides a window into the ecological processes underlying colonization success. Using a cross‐species and cross‐site dataset of wild‐caught invasive and native spider pairs, our comparative analysis revealed species‐ and location‐mediated differences in microbial diversity and composition. We observed lower endosymbiont abundance in invasive species, whereas we also observed site‐specific variability in endosymbiont in coexisting hosts. In contrast, native hosts exhibited higher endosymbiont diversity and abundance, though this pattern was highly location‐dependent, displaying an “all‐or‐nothing” distribution. Horizontally acquired gut microbiota was dominated by strong environmental filtering, with location acting as a stronger predictor of gut microbial community structure and abundance. However, species‐specific differences also drove patterns of diversity, indicating that although each spider may acquire a distinct microbial “fingerprint”, they also may differ in their host physiology and feeding behavior, thus driving species‐level variation.

Together, these findings underscore the dominant role of environmental factors and host identity in shaping microbial diversity and abundance in spiders. This highlights the importance of host‐specific habitat filtering and biogeography in shaping microbial communities in arthropods.

### 4.1 Environmental Filtering Dominates Gut Microbial Composition (H1)

Our results suggest a strong environmental filtering effect, supporting hypothesis 1 (H1), and in line with prior work showing that arthropod microbiota are often acquired through diet and environmental exposure (Paniagua Voirol et al. 2018; Zhang et al. 2021) and shift rapidly with changes in habitat or prey. Notably, even when the same spider species was sampled across different sites, gut microbial composition varied significantly, suggesting reassembly may occur each time the host encounters a novel microbial pool. PERMANOVA and pairwise PERMANOVA analysis consistently revealed that site‐level differences explained the largest proportion of variance across all species. Patterns of location‐specific effects on gut microbiota composition were not uniform across locations. Notably, specimens from the Berkeley site exhibited the least variance in gut microbiota composition, possibly reflecting reduced environmental heterogeneity or urban filtering effects. This exception highlights that although environmental filtering was a dominant force overall, its impact varied by habitat. The lack of consistency across sites in species‐specific variance indicates gut microbial communities in spiders are shaped predominantly by horizontal acquisition, meaning that microbes are picked up from the environment, including diet, rather than inherited from parent to offspring.

Importantly, a significant Site × Species interaction (*R* ^2^ = 0.066, *p* = 0.001) suggests that species‐specific differences in microbial communities vary by location, further supporting a model of context‐dependent host filtering. These results were further corroborated by SIMPER and DESeq2 analyses, which identified genera such as *Sediminibacterium* and *Propionibacterium* as key contributors to dissimilarity among sites, but not consistently among host taxa. ISA supported this trend, revealing microbial genera uniquely associated with specific environments, particularly on the Big Island and in Maui (Figure 1). Notably, *Sediminibacterium* and *Blastomonas* spp. also exhibited the highest predicted functional abundances, mirroring their elevated relative abundance in the gut (Figure S2). These results suggest that, even as each species and site assembles a unique taxonomic community, there may be convergent selection for key functional traits that support host physiology across diverse environments.

However, species identity also played a role in driving abundance—modulated by local environmental conditions—so gut communities reflect both who the host is and where it lives. Microbial communities on the Big Island, for example, formed distinct clusters across species, reinforcing the potential dominant role of local environmental filters in driving host‐specific patterns at specific sites. This pattern of context‐dependent species responses aligns with other arthropods, showing that hosts filter environmental pools in species‐specific ways (Zhang et al. 2021; Santos‐Garcia et al. 2020). The scale and consistency of these patterns challenge the notion that invasive spiders maintain a stable invasion‐associated microbiome across sites. Instead, our results reveal that each species reassembles its gut community according to its own niche and the local microbial reservoir, producing a mosaic of composition shaped by both host traits and habitat filtering.

### 4.2 Microbial Diversity Is Context Dependent, Not Uniformly Higher in Invaders (H2)

Our analyses of α‐diversity suggest that overall, the diversity of microbiomes in spider hosts varies significantly by species, but not by site or native/invasive classification, with no support for the hypothesis that invasion status is a major factor explaining microbial composition. β‐diversity analyses exhibited a different pattern from α‐diversity, with samples from the same site clustered together in NMDS plots. PERMANOVA echoed these results, indicating that site‐specific differences had a stronger effect than host species and native vs. invasive classification. Further, pairwise differences indicated a stronger difference between invasive and native species pairs, indicating that diversity may be site‐specific, but also relies on species‐level strategies for retaining diverse internal communities. Thus, although gut richness remains stable across sites, the identity of those microbes shifts with local environmental pools, such as diet, soil, and microhabitats, which may produce high turnover between islands. Contrary to hypothesis 2, invasive spiders did not sustain elevated within‐sample α‐diversity despite their generalist habits. Instead, each population's gut community appears to be assembled de novo from local microbial pools, illustrating that host physiology may set a baseline α‐diversity, whereas environmental filtering drives between‐sample β‐variation. Interestingly, *Tetragnatha* sp. from the Big Island showed significantly lower α‐diversity of gut microbes than other species at that site, potentially reflecting environmental stressors, although more data would be needed to test how environmental factors may vary with host fitness to impact microbial diversity.

### 4.3 Endosymbionts Absent or in Low Abundance in Invasive Species (H3)

We observed species–site specific “all‐or‐nothing” patterns rather than site‐wide trends, indicating microbial dominance at some sites, whereas others retained larger diversity. For example, Rickettsiella reached 100% prevalence in *S. grossa* on both Maui and Oʻahu, yet was completely absent from *S. grossa* on the Big Island. Conversely, Wolbachia was found consistently in *T. acuta* on the Big Island, whereas Rickettsiella was in all specimens of *T. eurychasma* on Oʻahu (Table S1). Other host–site combinations—such as *B. longinqua* in Berkeley (50% Wolbachia) or *T. versicolor* in California (46% Wolbachia)—fell into intermediate prevalence (20%–70%), underscoring that endosymbiont dominance could be a function of species‐specific responses to local filters, not uniform across all locations. However, infection rates below 30% are expected to be prey‐derived endosymbionts and thus cannot indicate true abundance shifts with respect to host filtering.

These patterns partially support H3's prediction of reduced symbiont abundance in invaders: invasive spiders often lack infections where co‐occurring natives carry them, whereas native Tetragnatha can approach fixation in select contexts. Comparable binary dynamics have been documented in Ariamnes spiders (Armstrong et al. 2021) and *Spiroplasma* in Drosophila (Jaenike et al. 2010), suggesting that long‐term co‐evolution and local environmental filtering jointly shape these tightly coupled host–symbiont associations. Thus, native species may maintain stronger or more specialized endosymbiont relationships, potentially because of longer co‐evolutionary histories or more stable environmental associations. Notably, the absence of *Wolbachia* and other common symbionts in *B. longinqua,* even in sites where they are prevalent in co‐occurring native spiders, suggests that these symbionts are not readily reacquired from the environment and may be filtered out because of host incompatibility or competitive exclusion. This challenges the idea that invasive species may be bringing microbial associates with them from ancestral habitats and instead suggests that different microbial strategies may aid adaptation to novel environments. However, because we lack native‐range data for *B. longinqua* (Simó et al. 2011), future work should sample its source populations across the invasion gradient to distinguish introduction‐driven shifts from species‐intrinsic traits.

Whether the low abundance of endosymbionts in invasive species constitutes a fitness benefit or detriment remains an open question. On the one hand, having fewer co‐dependent symbionts could reduce physiological constraints and allow for more flexible responses to novel environments. On the other hand, endosymbionts have known impacts on fitness, including host reproduction, sex ratios, and dispersal (Hu et al. 2019). Their low abundance in invasive hosts may limit these necessary functions, reflecting a potential cost to quick adaptation. Although this study provides proof of concept of the difference in endosymbiont‐host dynamics across sites and suggests a potential loss of endosymbiont functionality in invasive hosts, further study is needed to assess the mechanisms of these proposed fitness consequences.

### 4.4 Endosymbionts Reflect Host History (H4)

Unlike the gut microbiota, endosymbionts in this study showed stronger host‐specific patterns, albeit with striking variation in abundance. Native *Tetragnatha* species consistently carried multiple endosymbiont genera, including *Wolbachia*, *Rickettsia*, *Cardinium*, and *Rickettsiella*, often with high relative abundance and site specificity. In contrast, *B. longinqua* showed very low abundance of endosymbionts across all sites, and *S. grossa* displayed low and inconsistent presence, with no conserved taxa across sites. Dominant endosymbiotic taxa also emerged from our analysis, with *Wolbachia* and *Rickettsiella* abundant across all groups. The prevalence of these genera aligns with previous studies highlighting their diverse interactions with arthropods (Hien et al. 2022), with a Rickettsiella strain being recently shown to cause cytoplasmic incompatibility in a dwarf spider (Rosenwald et al. 2020). Our binomial GLMMs further demonstrated that endosymbiont abundance was only significant in select site–species combinations and most host–site pairs showed no significant differences, highlighting that overall symbiont prevalence does not differ broadly across species (Table 3). However, because endosymbionts occur at very low overall abundances relative to gut microbes, much of the variation across sites and species is necessarily muted, limiting our ability to detect significant differences in many comparisons.

Phylogenetic analysis of endosymbiont ASVs further revealed broadly intermingled lineages across both islands and host taxa, with a tendency for *Tetragnatha* ASVs from Hawaiian islands to cluster across native habitat sites. Importantly, Figure 2B shows that native Tetragnatha populations harbor higher ASV diversity within each species at a single site, which was not observed in invasive species patterns, underscoring the presence of a structured, multi‐lineage community in natives. Taken together, these results suggest that, whereas endosymbiont assemblages are largely shared across hosts and locations, native Tetragnatha maintain stronger within‐site phylogenetic structuring that reflects long‐standing host–symbiont associations.

Overall, these results support our last hypothesis (H4) that native species may contain more unique endosymbiont assemblages because of the development of symbiotic relationships over time, with tight coupling of endosymbionts to host phylogeny and vertical transmission (Hu et al. 2019; Armstrong et al. 2021), but also suggest that endosymbiont‐host relationships may be disrupted by adaptations to novel environments. However, because of the low abundance of many endosymbionts, future work could perform specific strain counts to improve endosymbiont detection across all site‐species combinations.

### 4.5 Ecological Implications

Our findings challenge the notion of a stable “invasion microbiome”—the hypothesis that successful invaders carry with them a core suite of microbial taxa that facilitate establishment in new habitats (Bonthond et al. 2021, 2023). Instead, invasive spiders appear to assemble gut microbial communities de novo at each site, shaped by local environmental filters. Although taxonomic identity shifts across habitats, certain gut microbiota are consistently retained. This could be a key axis of adaptability for generalist invaders, allowing them to colonize heterogeneous environments through strong microbial filtering. However, endosymbionts tell a different story. Their abundance, diversity, and phylogenetic consistency suggest longer‐term co‐evolution with native hosts, but their disruption in invaders highlights a potential shift in strategy for highly effective invasive species (Berlanga and Guerrero 2016).

### 4.6 Limitations and Future Directions

Our findings build on a growing literature that points to a strong role of environmental factors in shaping spider gut microbiomes and endosymbiotic partnerships, but several limitations should be addressed. First, sample sizes were limited at some sites, and sampling was not consistent across all islands. More fine‐scale sampling within islands would help clarify how environmental acquisition of microbes varies locally and how prey communities influence gut composition. Seasonal differences also posed a constraint. Collections from the Big Island and Berkeley were months apart, and because spider size and feeding behavior shift with life stage (Masumoto 1993; Anotaux et al. 2016), this may have introduced unwanted variation. Logistical constraints limited our capacity to sample across multiple islands at similar time periods, and thus, our study is limited by a potential seasonal contribution to our results. Future studies should coordinate sampling across time and space to reduce these effects and obtain samples of similar invasive species to improve comparisons across pairs and allow for three‐way statistical tests. In addition, gender identification and more specific species assignments (such as in Oahu *Tetragnatha*) would allow assessment of sex‐mediated morphological differences that could change microbial composition and diversity.

Importantly, for more detailed insights into the endosymbionts, starvation of spiders prior to sampling the microbiome would allow clearer separation of endosymbionts derived from the host versus those from prey. For functional predictions, improved microbial taxonomic resolution, especially for unclassified ASVs, will be essential. In addition, 16S rRNA amplicon sequencing provides only relative abundance estimates, which cannot directly distinguish colonization from transient DNA. In order to assess true endosymbiont colonization, targeted quantitative PCR (qPCR) would enable precise estimation of bacterial copy number per host biomass. This work would also aid in differentiating endosymbionts from gut microbiota, since narrowing our analysis to only five endosymbionts may limit symbiotic diversity assessments. Further, absolute‐abundance measurements will also overcome a key compositional limitation: an apparent increase in one taxon may simply reflect a decrease in another. Although we mitigated compositional bias via centered‐log‐ratio (CLR) transformation, direct bacterial load data are needed to confirm our findings. Thus, our study lays the groundwork for more cross‐species comparisons of endosymbiont colonization using enhanced experimental approaches. Linking microbial identity to host diet or physiology will help test whether changes in microbial composition influence host performance. Additionally, measuring invasion success more directly with parameters like range size, population growth, or dispersal rates could help evaluate whether microbial changes offer adaptive benefits.

Although the ecological consequences of microbiome variation in wild arthropods are still poorly understood, our study supports the idea that natural variation in microbial composition and diversity differs across native and invasive species, even in highly transient gut systems. Continued research is needed to uncover the functional roles of microbiomes across native and invasive taxa in changing environments.

## Author Contributions

**Madison J. Pfau:** conceptualization (lead), data curation (lead), formal analysis (lead), methodology (lead), project administration (equal), visualization (lead), writing – original draft (lead), writing – review and editing (equal). **Sven Weber:** conceptualization (supporting), data curation (supporting), formal analysis (supporting), methodology (supporting), visualization (supporting), writing – review and editing (equal). **Susan Kennedy:** data curation (supporting), project administration (supporting), resources (supporting), writing – review and editing (supporting). **Henrik Krehenwinkel:** conceptualization (supporting), data curation (supporting). **George Roderick:** funding acquisition (equal), project administration (supporting), resources (equal), supervision (supporting), writing – review and editing (supporting). **Rosemary Gillespie:** funding acquisition (equal), project administration (equal), supervision (equal), visualization (supporting), writing – review and editing (equal).

## Conflicts of Interest

The authors declare no conflicts of interest.

## Supporting information


**Appendix S1:** ece372175‐sup‐0001‐AppendixS1.docx.

## Data Availability

Raw data files and associated ASV tables for this project can be found on Dryad (DOI: 10.5061/dryad.bnzs7h4p4). Remaining scripts and data files used for downstream analysis can be found on the following public GitHub repository (https://github.com/madisonpf/invasive‐native‐spider‐microbiomes).
