# Supplementary material for: Invasive Spiders and Their Microbiomes: Patterns of Microbial Variation in Native and Invasive Species in Hawai'i
Source: Ecol Evol. 2025 Oct 2;15(10):e72175. doi: 10.1002/ece3.72175 (PMC12490970; doi:10.1002/ece3.72175)
Supplement: Supplementary file 1 — Appendix S1: ece372175‐sup‐0001‐AppendixS1.docx. [file ECE3-15-e72175-s001.docx]

Supplemental Materials for “**Invasive spiders and their microbiomes: Patterns of microbial variation in native and invasive species in Hawai‘i”**


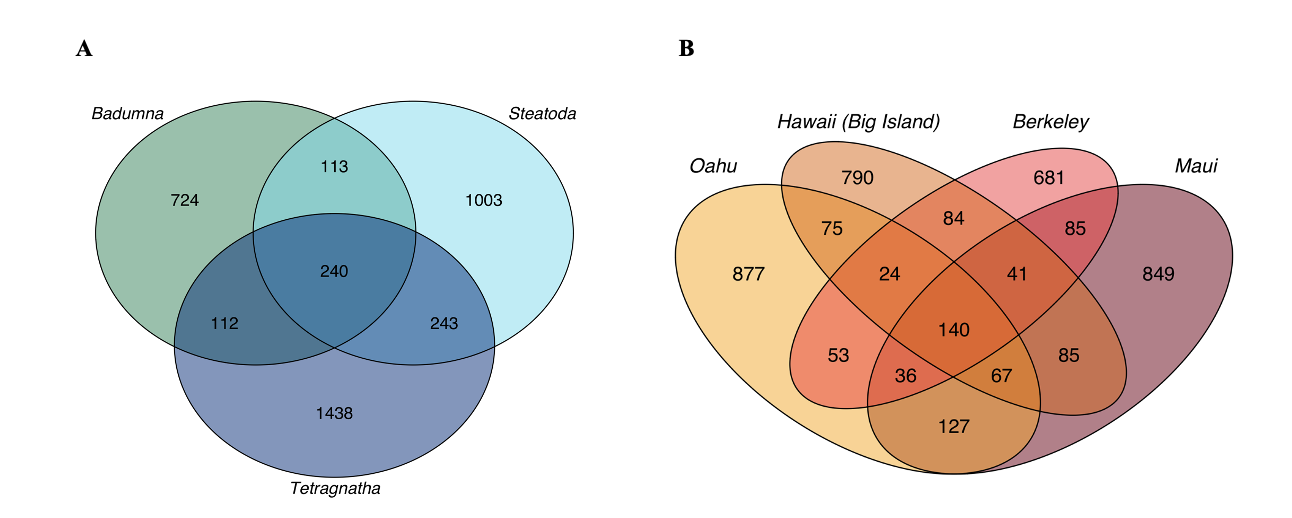
Figure 1. Venn Diagram plots depict the read distribution across host genera (A) and geographical location (B).

Table 1. Infection rates (proportion of individuals infected) for four endosymbiont genera across islands and spider species (N = number of specimens per group).


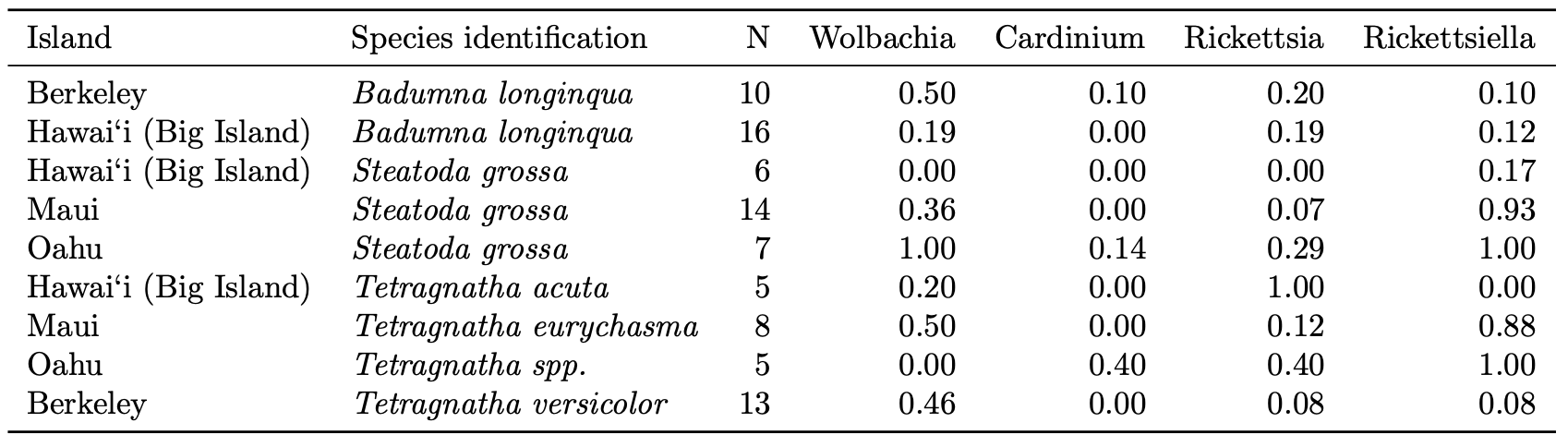


Table 2. Strain consistency of endosymbiont ASVs across islands and spider hosts: number of unique ASVs per genus and number of individuals sampled.


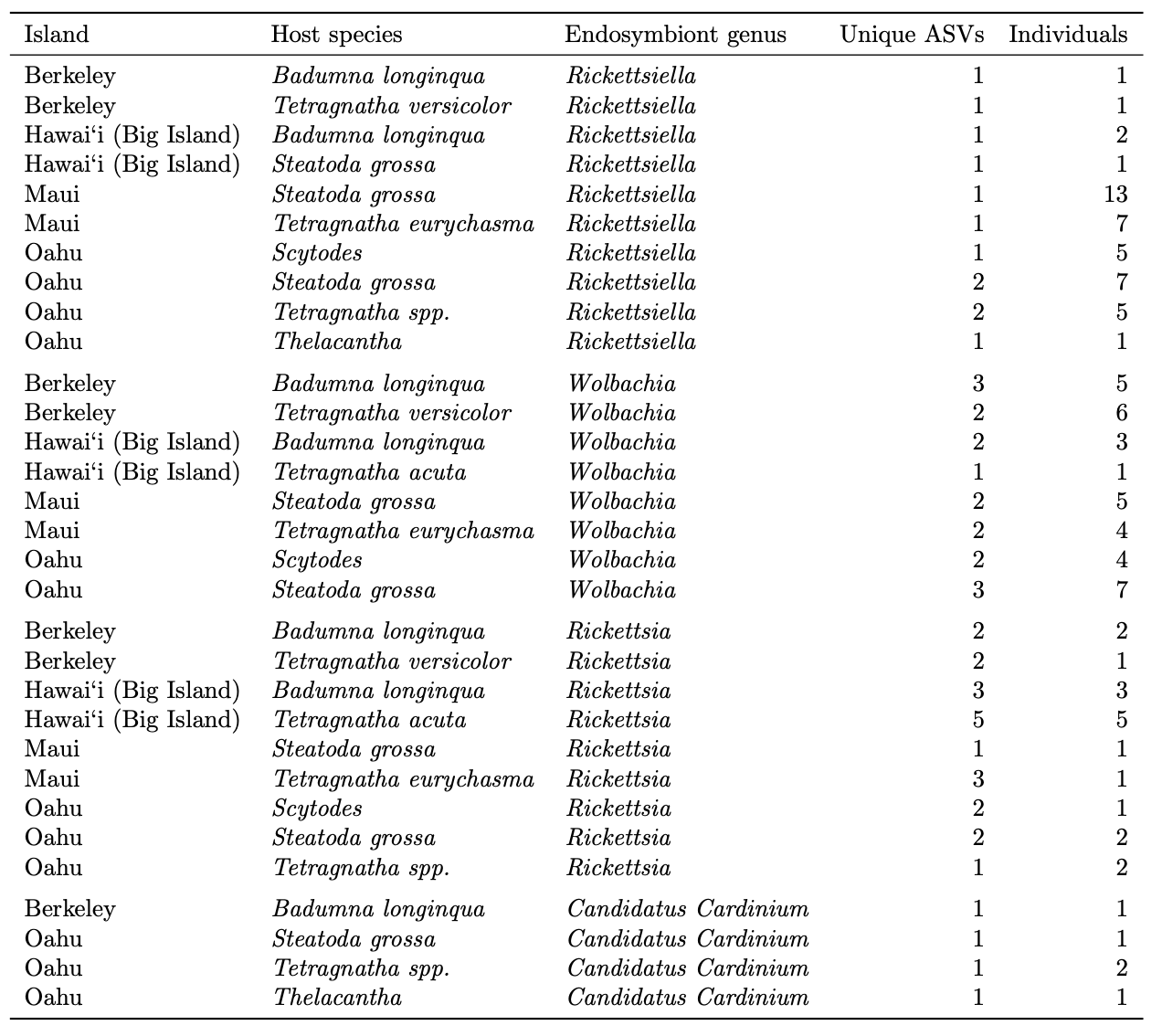


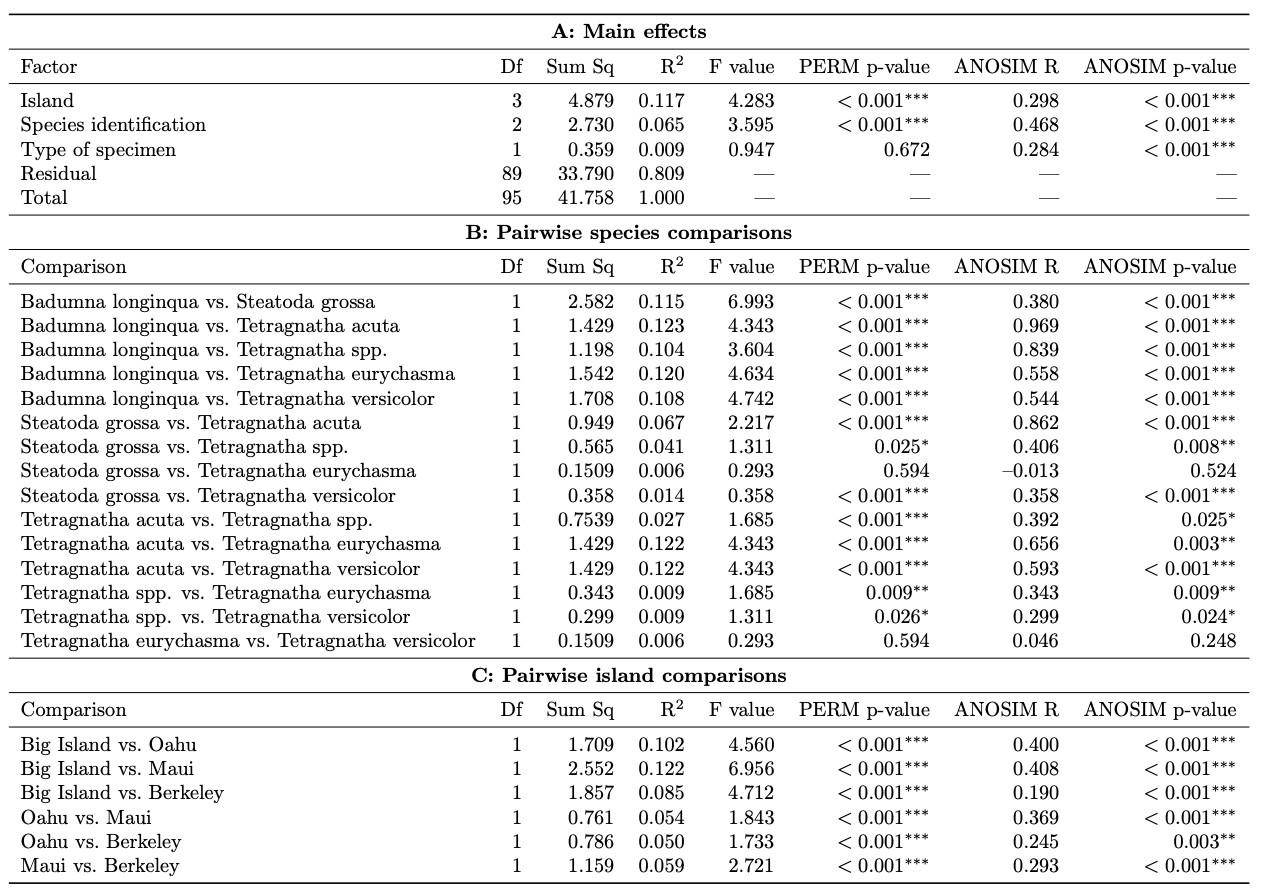
Table 3. PERMANOVA on Bray–Curtis dissimilarity of gut microbiota (β‑diversity; 999 permutations) showing main effects of Island, Species Identification, and Type of Specimen, followed by pairwise species and island comparisons. Analysis of similarities (ANOSIM) provided to justify PERMANOVA results.


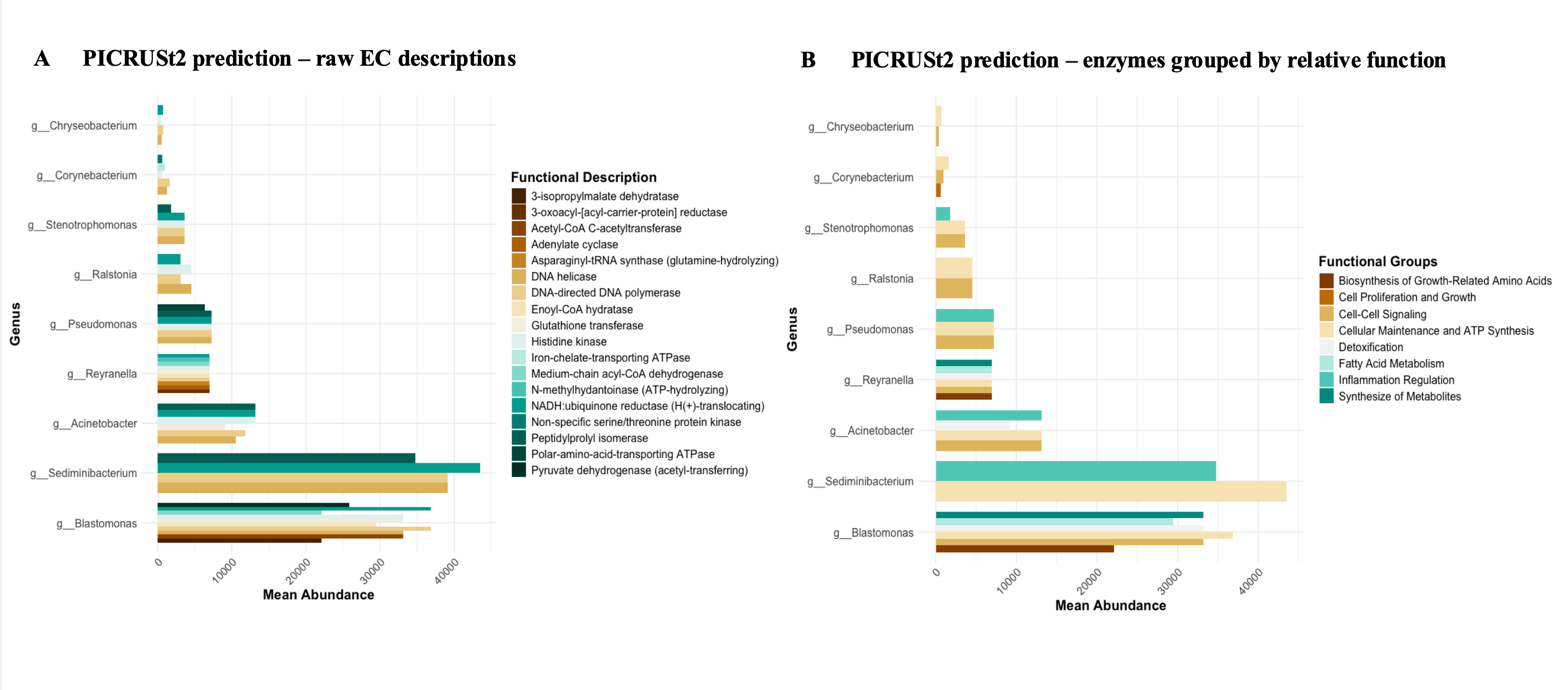


*Figure 2. Results of PICRUSt2 functional analysis showcasing top 10 functional gene pathways grouped by microbial genus. (A) illustrates the raw descriptions of functional gene pathways based on the EC METACYC enzymatic pipeline and the average taxonomic abundance of each function in each genus. (B) shows the same empirical data with enzymatic functions grouped by relative function corroborated by existing literature.*
